# Supplementary material for: Behavioral, neural and ultrastructural alterations in a graded-dose 6-OHDA mouse model of early-stage Parkinson's disease
Source: Sci Rep. 2023 Nov 9;13:19478. doi: 10.1038/s41598-023-46576-0 (PMC10636184; doi:10.1038/s41598-023-46576-0)
Supplement: Supplementary file 1 — Supplementary Figures. [file 41598_2023_46576_MOESM1_ESM.pdf]

Supplementary Material

Behavioral, neural and ultrastructural alterations in a graded-dose 6-OHDA mouse model of early-stage Parkinson's disease

Andrea Slézia[1,2,3,4*†], Panna Hegedüs[1,5,†], Evgeniia Rusina[2,†], Katalin Lengyel[1], Nicola Solari[1], Attila Kaszas[4], Diána Balázsfi[1], Boris Botzanowski[2], Emma Acerbo [2], Florian Missey[2], Adam Williamson[2,6*] and Balázs Hangya[1*]

1. Lendület Laboratory of Systems Neuroscience, Institute of Experimental Medicine, Budapest, Hungary

2. Institut de Neurosciences des Systèmes, INSERM UMR S 1106, Aix-Marseille Université, Marseille, France

3. Institute of Cognitive Neuroscience & Psychology, Eotvos Lorand Research Network, Budapest, Hungary

4. Institut de Neurosciences de la Timone, CNRS UMR 7289, Aix-Marseille Université, Marseille, France

5. János Szentágothai Doctoral School of Neurosciences, Semmelweis University, Budapest, Hungary

6. International Clinical Research Center (ICRC), St. Anne's University Hospital, Brno, Czech Republic

* Correspondence: Andrea Slézia (andrea.slezia@gmail.com), Adam Williamson (adam.williamson@fnusa.cz) and Balázs Hangya (hangya.balazs@koki.hu)

Supplementary Figure S1

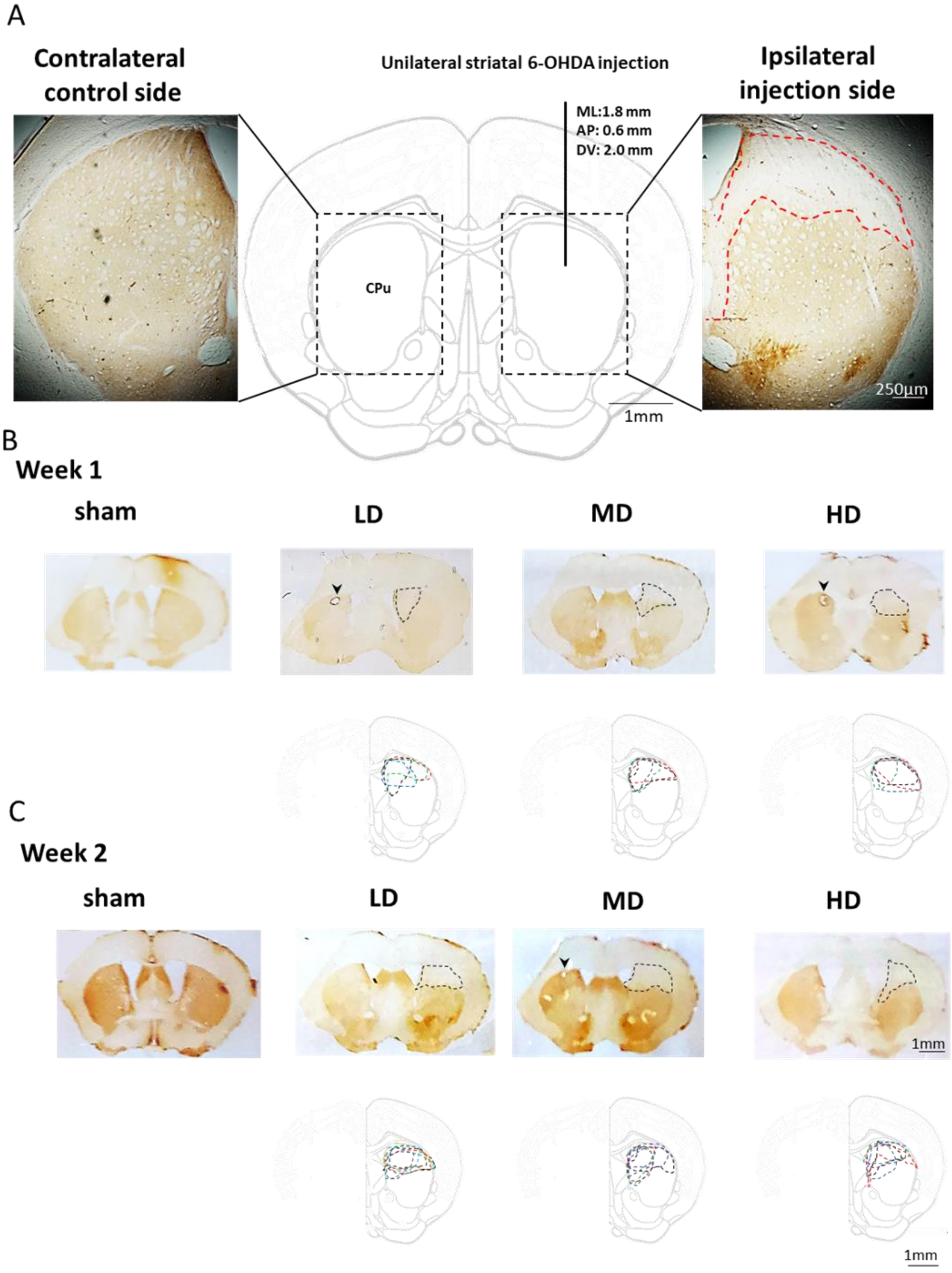

Supplementary Figure S1. Injection sites. A, A representative example of a striatal high-dose 6-OHDA injection following two weeks of survival. Light micrographs of coronal sections are shown. B, Top, representative examples from sham-operated, LD, MD and HD mice one week after injections. Bottom, lesion sites demonstrated on reference mouse brain atlas coronal sections. Arrows show tissue marks of the contralateral side of the blocks of mouse brains. Tangential cuts of the cortex contralateral to the lesions also served as side markers. C, Same as in panel B but two weeks after injections. The figure was created with BioRender.com.
